# Prevalence of Rest Intolerance and Its Associations With Mental Health and Quality of Life Among Adults in China: Cross-Sectional Study

**DOI:** 10.2196/100611

**Published:** 2026-10-01

**Authors:** Fei Wang, Xuanjing Ji, Xin Guan, Ting Wang, Rui Zhong, Yibo Wu, Tingshao Zhu

**Affiliations:** 1 State Key Laboratory of Cognitive Science and Mental Health, Institute of Psychology, Chinese Academy of Sciences Beijing China; 2 Department of Psychology, University of Chinese Academy of Sciences Beijing China; 3 School of Public Health, Shandong University Jinan China; 4 School of Nursing, China Medical University Shenyang China; 5 School of Nursing, Jilin University Changchun China; 6 School of Psychology, South China Normal University Guangzhou China; 7 Department of Nursing, the Fourth Affiliated Hospital of School of Medicine, and International School of Medicine, International Institutes of Medicine, Zhejiang University Yiwu China

**Keywords:** rest intolerance, quality of life, mental health, prevalence, Chinese adults

## Abstract

**Background:**

Rest intolerance, the experience of negative emotions when attempting to rest, is an emerging public health concern, yet its population-based epidemiology remains poorly understood.

**Objective:**

This study systematically investigated the prevalence, distribution, and health correlates of rest intolerance among adults in China, with particular focus on its manifestation in highly educated and younger populations.

**Methods:**

We analyzed cross-sectional data from 24,405 adults in the 2024 Psychology and Behavior Investigation of Chinese Residents (PBICR) survey. Rest intolerance (Rest Intolerance Scale–8 [RIS-8]), mental health (Patient Health Questionnaire–9 [PHQ-9], Generalized Anxiety Disorder–R3 [GAD-R3], and Perceived Stress Scale–4 [PSS-4]), and quality of life (EQ-5D-5L or EuroQol visual analog scale [EQ-VAS]) were assessed. Cutoffs were derived via cluster analysis and latent profile analysis, followed by regression and moderation analyses.

**Results:**

The overall prevalence of high rest intolerance was 14.94% (3645/24,405). Notably, rates were significantly elevated among younger adults and those with higher educational levels. Rest intolerance was significantly associated with adverse mental health outcomes (anxiety: β=0.406; depression: β=0.402; and perceived stress: β=0.440; all *P*<.001) and reduced quality of life (EQ-5D: β=−0.115 and EQ-VAS: β=−0.117; both *P*<.001). Moderation analyses further revealed that these adverse associations were stronger among highly educated and younger individuals.

**Conclusions:**

Rest intolerance affects 14.94% of adults in China, with highly educated and younger populations constituting particularly high-risk groups. The condition demonstrates robust associations with adverse mental health outcomes and diminished quality of life, especially among these populations. These findings underscore the need for targeted psychological interventions and workplace health policies addressing rest-related distress in contemporary performance-oriented societies.

## Introduction

In today’s fast-paced and high-pressure society, the fundamental physiological and psychological need for rest faces unprecedented challenges [1]. For many individuals, rest is no longer a simple act of relaxation and recovery; instead, it may evolve into a source of anxiety and discomfort [2]. This phenomenon, termed “rest intolerance,” was first identified and operationalized through empirical research on college students who reported feeling unable to psychologically disengage from work or study during breaks, experiencing guilt, rumination, and social comparison even when physically at rest [3]. Clinically, rest intolerance is characterized by 4 core features: negative affect (eg, guilt and anxiety during rest), cognitive bias (viewing rest as wasteful or dangerous), obsessive thoughts about unfinished tasks, and heightened social comparison regarding productivity [3]. It is critical to distinguish rest intolerance from conventional sleep disorders: although sleep disorders primarily involve physiological dysregulation of sleep-wake cycles, rest intolerance is a psychological construct centered on the inability to experience rest as restorative due to internalized performance pressures. An individual may sleep adequately yet still experience rest intolerance if psychological distress occurs during waking rest periods. Emerging evidence indicates that rest intolerance is associated with a range of adverse outcomes, including elevated anxiety, depressive symptoms, perceived stress, diminished quality of life, and even problematic smartphone use as a maladaptive avoidance strategy [4-8].

Despite its growing recognition, research on rest intolerance remains in an early stage. As the construct was only recently formally operationalized [3], existing studies have largely relied on small, homogeneous samples—primarily university students [9,10] or specific occupational groups such as nursing interns [8] and athletes [11]—limiting the generalizability of findings. No large-scale epidemiological survey has yet established population-level prevalence rates, validated screening thresholds, or systematically examined demographic distribution patterns. This gap is particularly concerning given the construct’s public health relevance: if rest intolerance affects substantial segments of the population and exacerbates mental health burdens, identifying at-risk groups and quantifying its health impacts is essential for designing targeted psychological interventions and workplace health policies. Addressing rest intolerance aligns with broader public health objectives of reducing the societal burden of anxiety and depression, promoting genuine psychological recovery, and fostering sustainable work-life balance in performance-oriented societies.

The performance internalization model of rest intolerance posits that in highly performance-oriented sociocultural environments, individuals internalize beliefs such as “effort equals virtue” and “output equals value” as core criteria for self-identity through family upbringing, examination-oriented education, and social conditioning [12]. This internalization process triggers automatic self-critical thoughts during rest, directly inducing shame and guilt. Critically, this process may be particularly pronounced among individuals whose self-worth is highly contingent upon performance in these internalized domains [13]. Simultaneously, under intense social comparison pressures, which are further amplified by cultural norms that signal busyness and a lack of leisure as markers of higher status [14], individuals perceive threats of falling behind through direct observation or imagined monitoring, further framing rest as a dangerous and immoral act. Compounded by the sense of time loss and unfinished task anxiety stemming from high-intensity workloads, rest is experienced as a betrayal of responsibility and goals. When individuals overly rely on external validation to confirm their self-worth, rest becomes more easily interpreted as a failure to meet expectations or an invitation to negative labeling. Through the interplay of these mechanisms, rest ceases to be a restorative experience and instead evolves into a systemic psychological predicament [12].

On the basis of this theoretical framework, this study posits that educational attainment and age are key factors shaping the risk of rest intolerance. Individuals with higher education have undergone longer and more systematic examination-oriented education and academic discipline, exhibiting deeper internalization of performance-oriented values and greater dependence on external evaluation. Consequently, they are expected to exhibit higher levels of rest intolerance. Younger individuals, navigating the transition from school to the workplace, face intense task demands and time management pressures while exhibiting heightened sensitivity to social comparison signals. They are more likely to perceive rest as a threat to self-identity, leading to significantly higher rest intolerance levels than those of middle-aged and older adults. Within the Chinese social context, this issue may exhibit distinctive distribution patterns [15]. With the expansion of higher education and intensifying social competition, highly educated individuals often harbor greater career expectations and work commitment, potentially leading to more complex perceptions and attitudes toward nonproductive rest time [16,17]. Younger individuals, positioned at the beginning or ascending phase of their careers, face greater achievement pressures and time constraints, making them more susceptible to rest intolerance [18,19]. Therefore, we have reason to hypothesize that highly educated and younger individuals may constitute high-risk groups for rest intolerance. However, this hypothesis remains unsupported by large-scale epidemiological survey data.

To address these gaps, the present study leveraged a nationally representative sample of 24,405 adults in China to (1) establish robust screening thresholds for rest intolerance via cross-validation of cluster analysis and latent profile analysis; (2) estimate the population prevalence and demographic distribution; and (3) systematically examine its associations with mental health and quality of life, with particular focus on whether educational attainment and age amplify these adverse effects. By clarifying these issues, this study aimed to enhance public and academic awareness of rest intolerance, particularly by drawing attention to the psychological welfare of high-risk groups. This research provides scientific evidence for developing targeted psychological interventions and health promotion strategies in the future.

## Methods

### Study Population

Data for this study were derived from the Psychology and Behavior Investigation of Chinese Residents (PBICR) survey, conducted between June and September 2024. This cross-sectional survey covered 22 provinces, 5 autonomous regions, 4 municipalities directly under the central government, and 2 special administrative regions in China [20,21]. Given that this study aimed to examine differences in psychological and behavioral characteristics across demographic subgroups, a single sampling method would have been insufficient to simultaneously ensure representativeness and control for sample structure. Therefore, a multistage sampling strategy combining stratified random sampling and quota sampling was used. The sampling strategy served a dual purpose: achieving broad geographic and urban-rural representation at the macro level while ensuring that key demographic characteristics (eg, gender and age) reflected those of the target population at the micro level, thereby reducing selection bias. Specifically, the sampling process was implemented in 3 distinct stages. In the first stage, cities were selected based on population size and economic and cultural considerations within each province or autonomous region. Using a random number table, 2 to 12 cities were drawn from the aforementioned areas, resulting in a total of 150 cities included in the sample. The primary objective of this stage was to ensure representation across regions with varying levels of development and diverse social environments. In the second stage, the number of communities selected within each city was determined based on the population size of the corresponding administrative region. From each city, 10 to 60 communities were drawn using a 3:2 urban-to-rural ratio, resulting in a total of 800 communities. This stage was designed to balance the urban-rural distribution and prevent oversampling in either urban or rural areas. In the third stage, quota sampling was used to select residents within each community. Quotas were set to achieve a 1:1 gender ratio and an age distribution approximately aligned with the age structure of China’s population pyramid [22]. This quota control was primarily intended to mitigate demographic structural bias arising from voluntary participation or differential on-site accessibility, thereby enhancing the comparability of the sample across gender and age dimensions. During data collection, trained investigators conducted one-on-one interviews and administered face-to-face questionnaires to each participant on site. This approach served 2 purposes: first, to reduce measurement error introduced by comprehension difficulties, variations in reading ability, or careless responding; and second, to enhance questionnaire completion rates and data integrity for key variables, an advantage particularly relevant to surveys involving psychological perceptions and subjective experiences. The entire study protocol was subject to standardized quality control procedures and was registered with the Chinese Clinical Trial Registry (ChiCTR) under registration number ChiCTR2400085016. After data cleaning, which excluded questionnaires with excessively short completion times, patterned responses, logical inconsistencies, or missing data on key variables, a final sample of 24,405 valid responses was obtained.

### Measurement Instruments

#### Sociodemographic Questionnaire

A self-designed sociodemographic questionnaire was used to collect basic demographic information from participants, which served as covariates and control variables in the empirical analyses. Following standard practices in previous research on mental health and quality of life, this study included 4 core demographic variables: gender, age, educational attainment, and ethnicity [23]. Age was categorized into 3 groups according to conventional demographic classifications: young adults (aged 18-34 years), middle-aged adults (aged 35-59 years), and older adults (aged ≥60 years). Educational attainment was classified into 8 levels: primary school, junior middle school, secondary specialized school, senior high school, junior college, undergraduate, master’s degree, and doctoral degree. Ethnicity was categorized as Han or minority ethnic groups.

#### Quality of Life Scale

Health-related quality of life was assessed using the EQ-5D-5L and the EuroQol visual analog scale (EQ-VAS) [24]. Developed by the World Health Organization, the EQ-5D-5L covers 5 dimensions of health: mobility, self-care, usual activities, pain or discomfort, and anxiety or depression. Each dimension is rated on 5 levels of severity, ranging from “no problems” to “extreme problems.” The EQ-VAS serves as a complementary tool, asking respondents to rate their overall health on a scale from 0 (“the worst health you can imagine”) to 100 (“the best health you can imagine”). In this study, the Cronbach α coefficient of the EQ-5D-5L was 0.86.

#### Rest Intolerance Scale–Short Form

The short form of the Rest Intolerance Scale–8 (RIS-8), developed and validated by Wang et al [3], was used in this study. The scale consists of 8 items across 4 dimensions: negative affect, cognitive bias, obsessive thoughts, and social comparison. Each item is rated on a 5-point Likert scale. Total scores range from 8 to 40, with higher scores indicating greater rest intolerance. In the present study, the Cronbach α coefficient for the overall RIS-8 was 0.93, and the coefficients for the 4 subscales ranged from 0.76 to 0.89, all exceeding the acceptable threshold of 0.60, indicating good reliability and validity. It should be noted that, given that the RIS-8 was initially developed in a university student population, we further examined its reliability, validity, and measurement invariance in the general population. The results confirmed that the RIS-8 demonstrated good reliability and construct validity, with measurement invariance established across gender, age, educational attainment, and ethnicity. For detailed information, please refer to Multimedia Appendices 1 and 2.

#### Mental Health Assessment Scales

Mental health status was assessed using the Patient Health Questionnaire–9 (PHQ-9) [25], the Generalized Anxiety Disorder–R3 (GAD-R3) [26], and the Perceived Stress Scale–4 (PSS-4) [27]. The PHQ-9 consists of 9 items rated on a 4-point scale ranging from 0 (“not at all”) to 3 (“nearly every day”), with total scores ranging from 0 to 27. A score of ≥10 indicates moderate to severe depressive symptoms. The Cronbach α coefficient of the PHQ-9 in this study was 0.94. The GAD-R3 comprises 3 items, also rated on a 4-point scale. The Cronbach α coefficient of the GAD-R3 in this study was 0.90. The PSS-4 is a 4-item short-form version of the Perceived Stress Scale. Items are rated on a 5-point scale ranging from 1 (“never”) to 5 (“very often”), with total scores ranging from 4 to 20. Higher scores indicate greater perceived stress. This version retains the core structure of the original scale and has demonstrated good reliability and validity, with Cronbach α exceeding 0.93.

### Statistical Analysis

First, to precisely identify high-risk individuals, we combined cluster analysis and latent profile analysis to establish thresholds for rest intolerance. Using k-means clustering (optimized by the elbow rule) and latent profile analysis (with the Bayesian information criterion [BIC] used to select the best-fitting model), we separately identified groups with high rest intolerance. Subsequently, cutoffs were calculated using the nearest neighbor midpoint method, and the final threshold was determined by integrating results from both approaches. The k-means approach yielded a cutoff of 28, while the latent profile analysis yielded a cutoff of 29. Given that the 2 methods yielded highly consistent cutoffs (28 and 29), indicating convergence in classification, we adopted the average of the 2 values (28.5) to balance the respective strengths of each method rather than privileging one over the other. Given that the scale produces only integer values, this average was rounded down to 28 for practical application. On the basis of this threshold, all participants were categorized into high and nonhigh rest intolerance groups (ie, scores of ≥29 were classified as high rest intolerance, whereas scores of ≤28 were classified as nonhigh) to calculate the overall prevalence of high rest intolerance among adults in China.

Building on this foundation, the study focused on examining the differences in distribution of rest intolerance across distinct populations. We used ANOVA and independent-sample *t* tests (2-tailed) to systematically compare rest intolerance scores across age groups, educational attainment levels, gender, and ethnicity, followed by post hoc tests. On the basis of prior theoretical assumptions, we specifically examined whether younger individuals and those with higher educational attainment exhibited higher levels of rest intolerance.

To further elucidate the negative health associations of rest intolerance, we constructed a series of linear regression models. After controlling for confounding factors, including age, gender, educational attainment, and ethnicity, we tested the independent associations of total rest intolerance scores with health outcomes. Quality of life and mental health indicators served as dependent variables.

Finally, to validate the core hypothesis that educational attainment and age amplify the adverse associations of rest intolerance, we conducted moderation analyses. We separately examined the moderating effects of educational attainment and age on the relationship between rest intolerance and both quality of life and mental health, aiming to provide deeper empirical evidence for targeted interventions among highly educated and younger populations.

All statistical analyses were conducted using R (version 4.3.0; R Foundation for Statistical Computing), with a significance level set at α=.05.

### Ethical Considerations

This quantitative study was performed in accordance with the Ministry of Health Involves People in Biomedical Research Ethics Review Method trial, the National Medical Products Administration’s Standard for Quality Control of Drug Clinical Trials (2003), the Medical Device Clinical Trial Regulations (2004), and the Declaration of Helsinki. PBICR 2024 was approved by the ethics committee of Shanghai Jiao Tong University (H20240237I). We certify that all applicable institutional and governmental regulations concerning the ethical use of human volunteers were followed over the course of this research. All interviewees provided electronic informed consent to participate in this study upon recruitment. Participants had the opportunity to receive a small gift from the research team.

## Results

### Sociodemographic Characteristics of the Sample

The basic characteristics of the sample are presented in Table 1. Among the participants, 49.2% (12,010/24,405) were men, and 50.8% (12,395/24,405) were women. Middle-aged adults constituted the largest age group, accounting for 45.7% (11,155/24,405) of the sample. Most participants had attained an undergraduate education, and the sample was predominantly composed of individuals of Han ethnicity.

**Table 1:** Sociodemographic information of the sample (N=24,405).

Variables			Participants, n (%)
**Gender**			
	Men	12,010 (49.2)	
	Women	12,395 (50.8)	
**Age group (years)**			
	18-34 (young adults)	9792 (40.1)	
	35-59 (middle-aged adults)	11,155 (45.7)	
	≥60 (older adults)	3458 (14.2)	
**Educational attainment**			
	No formal education	1127 (4.6)	
	Primary school	2016 (8.3)	
	Junior middle school	3554 (14.6)	
	Secondary specialized school	1759 (7.2)	
	Senior high school	3165 (13)	
	Junior college	3669 (15)	
	Undergraduate	7825 (32.1)	
	Master’s degree	1012 (4.2)	
	Doctoral degree	278 (1.1)	
**Ethnicity**			
	Han ethnicity	22,490 (92.1)	
	Minority ethnic group	1915 (7.9)	

### Cutoffs for Rest Intolerance Among Adults in China

Cluster analysis was used to establish cutoffs for rest intolerance among adults in China. Specifically, the elbow method was used to visualize the within-cluster sum of squares across different numbers of clusters, which indicated that a 4-cluster solution was optimal. Subsequently, k-means clustering was performed with the optimal number of clusters. As shown in Table 2, cluster 4 exhibited the highest mean rest intolerance score and was therefore designated as the high rest intolerance group. Cutoffs were derived by calculating the midpoint between the maximum value of the lower adjacent class and the minimum value of the higher adjacent class. On the basis of this method, the cutoff for rest intolerance among adults in China was determined to be 28.

**Table 2:** Cluster analysis results (N=24,405).

Clusters	Score, mean (SD)	Score, median (IQR)	Scores, range	Participants, n (%)
Cluster 1	9.46 (1.84)	8 (3)	8-14	3990 (16.35)
Cluster 2	17.4 (2.04)	17 (3)	13-22	6968 (28.55)
Cluster 3	24.1 (1.91)	24 (2)	19-29	9716 (39.81)
Cluster 4	32.5 (3.29)	32 (4)	27-40	3731 (15.29)

To ensure the robustness of the cutoffs, latent profile analysis was also conducted to derive cutoffs for the RIS-8. Specifically, fit indices indicated that a 6-class solution provided the best model fit. Class 6 was identified as the high-risk group for rest intolerance, with total scores ranging from 27 to 40. Detailed results of the fit indices for each category are presented in Multimedia Appendix 3. Following a similar approach to that used in the cluster analysis, the cutoff was derived by calculating the midpoint between the maximum value of the lower adjacent class and the minimum value of the higher adjacent class. On the basis of this method, the cutoff for rest intolerance among adults in China was determined to be 29, as illustrated in Figure 1.

Synthesizing the results from both cluster analysis and latent profile analysis, the average of the 2 cutoffs was 28.5. Given that the RIS-8 yields integer scores, the cutoff was accordingly set at 28. Participants with scores greater than 28 were classified as exhibiting high levels of rest intolerance.

**Figure 1:**
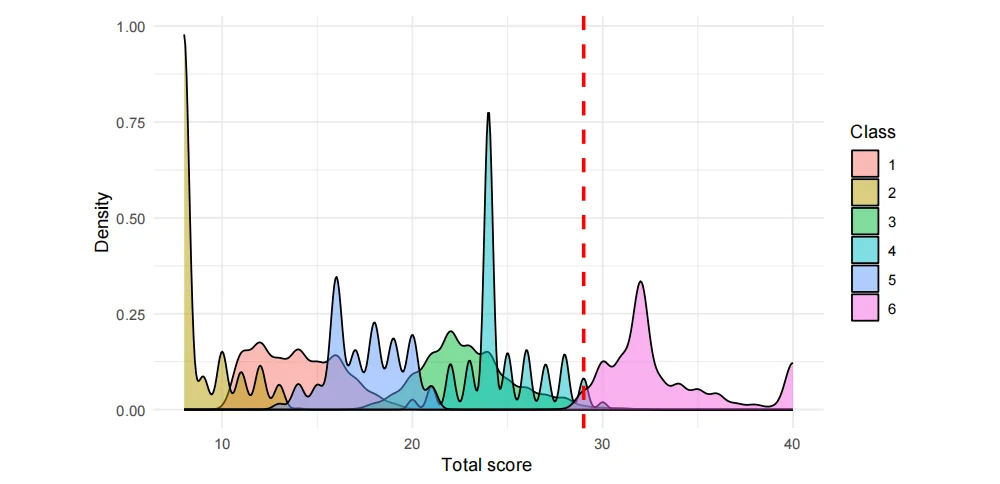
Sample distribution of Rest Intolerance Scale–8 (RIS-8) scores across latent profiles.

### Prevalence and Distribution of Rest Intolerance Among Adults in China

Using the cutoff established in the previous step, participants were classified into high rest intolerance and nonhigh rest intolerance groups. The overall prevalence of high rest intolerance among adults in China was found to be 14.94% (3645/24,405). Differences in both the prevalence of high rest intolerance and mean rest intolerance scores were further examined across age groups, gender, ethnicity, and educational attainment levels. Regarding the prevalence of high rest intolerance, prevalence rates increased significantly with higher levels of educational attainment, as illustrated in Figure 2.

**Figure 2:**
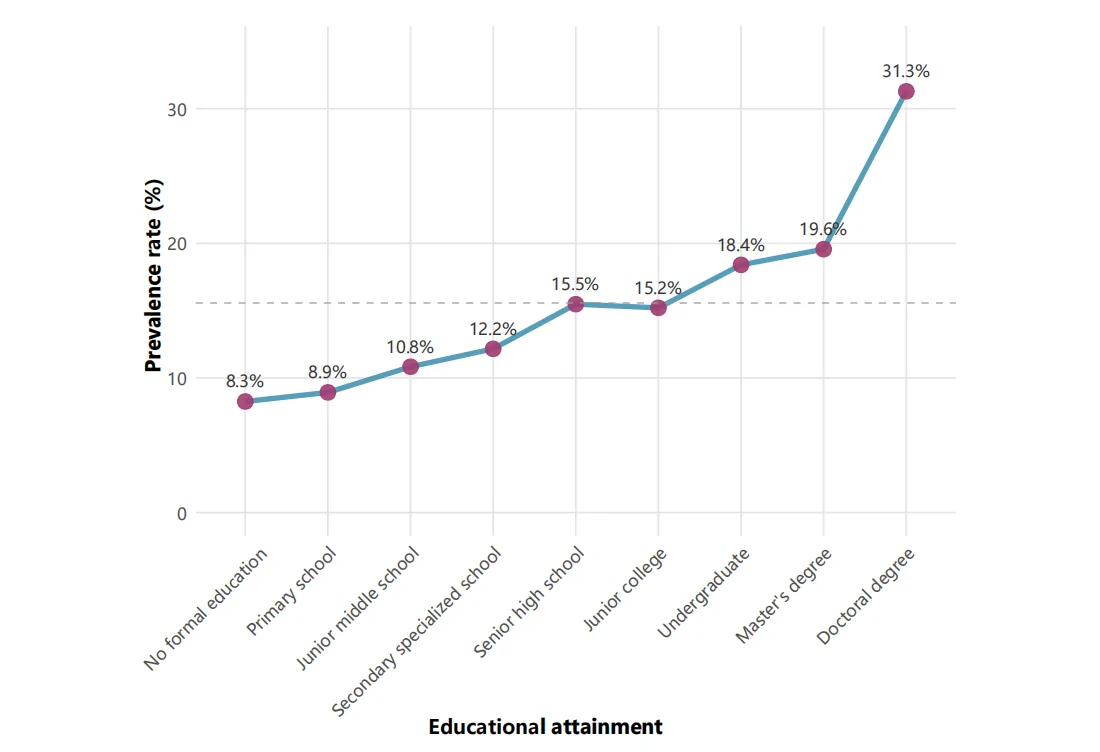
Prevalence of High rest intolerance by Educational Attainment.

ANOVA revealed significant differences in total rest intolerance scores across age groups (*F*_2,24,402_=403.9; η²=0.032; *P*<.001). Post hoc tests further indicated that older adults had significantly lower rest intolerance scores than both young adults (*P*<.001) and middle-aged adults (*P*<.001), while young adults scored significantly higher than middle-aged adults (*P*<.001). Significant differences in rest intolerance scores were also observed across educational attainment levels (*F*_8,24,396_=61.72; η²=0.020; *P*<.001). Post hoc comparisons revealed that, compared to the no formal education group, all other educational groups had significantly higher rest intolerance scores (*P*<.001), with the exception of the primary school group, which did not differ significantly (*P*=.97). Additionally, men reported significantly higher rest intolerance scores than women (t_24,268_=3.26; Cohen *d*=0.042; *P*=.001), while participants of Han ethnicity had significantly lower scores than participants from minority ethnic groups (t_2275.3_=−9.06; Cohen *d*=−0.210; *P*<.001). Detailed results are presented in Table 3.

**Table 3:** Distribution of total rest intolerance scores and prevalence of high rest intolerance (N=24,405).

Variables and subgroups		Prevalence of high rest intolerance, % (n/N)	Rest Intolerance Scale–8 score, mean (SD)	*t* test *(df*)		*F* test (*df*)		*P* value	
**Gender**					3.26 (24,268)		—^a^		.001
	Men	15.62 (1876/12,120)	21.24 (7.55)						
	Women	14.27 (1769/12,395)	20.94 (7.23)						
**Age group (years)**					—		403.9 (2, 24,402)		<.001
	18-34 (young adults)	18.68 (1829/9792)	22.43 (7.30)						
	35-59 (middle-aged adults)	13.13 (1492/11,359)	20.70 (7.20)						
	≥60 (older adults)	9.96 (324/3254)	18.40 (7.43)						
**Educational attainment**					—		61.72 (8, 24,396)		<.001
	No formal education	8.25 (93/1127)	18.93 (7.26)						
	Primary school	8.93 (180/2016)	19.24 (7.19)						
	Junior middle school	10.83 (385/3554)	20.01 (7.16)						
	Secondary specialized school	12.17 (214/1759)	20.84 (6.88)						
	Senior high school	15.48 (490/3165)	21.45 (7.32)						
	Junior college	15.21 (558/2669)	21.19 (7.52)						
	Undergraduate	18.4 (1440/7825)	22.02 (7.37)						
	Master’s degree	19.57 (198/1012)	21.84 (7.47)						
	Doctoral degree	31.29 (87/278)	23.94 (8.32)						
**Ethnicity**					−9.06 (2275.3)		—		<.001
	Han ethnicity	14.6 (3284/22,490)	20.97 (7.40)						
	Minority ethnic groups	18.85 (361/1915)	22.51 (7.17)						

^a^Not applicable.

### Associations Between Rest Intolerance, Quality of Life, and Mental Health

To examine the association between rest intolerance and quality of life, linear regression models were constructed with rest intolerance as the independent variable, adjusting for age, gender, educational attainment, and ethnicity. The results showed that total rest intolerance scores were significantly and negatively associated with both the utility index of quality of life (β=−0.115, 95% CI −0.128 to −0.102; *P*<.001) and EQ-VAS ratings (β=−0.117, 95% CI −0.130 to −0.105; *P*<.001). Furthermore, to examine the relationship between rest intolerance and mental health, separate linear regression models were constructed with anxiety, depression, and perceived stress as outcomes, again adjusting for age, gender, educational attainment, and ethnicity. The results indicated that rest intolerance was significantly and positively associated with levels of anxiety (β=0.406, 95% CI 0.394-0.418; *P*<.001), depression (β=0.402, 95% CI 0.390-0.414; *P*<.001), and perceived stress (β=0.440, 95% CI 0.428-0.451; *P*<.001). The stability analysis results are presented in Multimedia Appendix 4. Even after controlling for chronic disease status, smoking, and alcohol consumption, the results remained consistent.

### Moderating Effects of Educational Attainment and Age

We further examined whether educational attainment and age moderated the relationships between rest intolerance, quality of life, and mental health, after adjusting for other covariates. Detailed results are presented in Figure 3. The results indicated that educational attainment significantly moderated the associations between rest intolerance and both quality of life (EQ-5D: t_24,399_=−2.28; *P*=.02; EQ-VAS: t_24,399_=−4.01; *P*<.001) and mental health (depression: t_24,399_=7.67; *P*<.001; anxiety: t_24,399_=7.96; *P*<.001; perceived stress: t_24,399_=8.39; *P*<.001). Specifically, participants with higher educational attainment exhibited stronger associations between rest intolerance and both mental health and quality of life. Similarly, age significantly moderated the relationships between rest intolerance, quality of life, and mental health. Younger participants demonstrated stronger associations between rest intolerance and EQ-VAS (t_24,399_=6.15; *P*<.001), depression (t_24,399_=−12.30; *P*<.001), anxiety (t_24,399_=−12.75; *P*<.001), and perceived stress (t_24,399_=−12.10; *P*<.001). Negative *t* values reflect the coding of age as a continuous variable, with stronger associations observed at younger ages. However, no significant moderating effect of age was found on the relationship between rest intolerance and EQ-5D-5L (t_24,399_=0.95; *P*=.34). The stability analysis results are presented in Multimedia Appendix 4. Even after controlling for chronic disease status, smoking, and alcohol consumption, the main findings remained largely consistent, with 1 exception: the moderating effect of educational attainment on the association between rest intolerance and the EQ-5D-5L utility index became nonsignificant. All other results remained unchanged.

**Figure 3:**
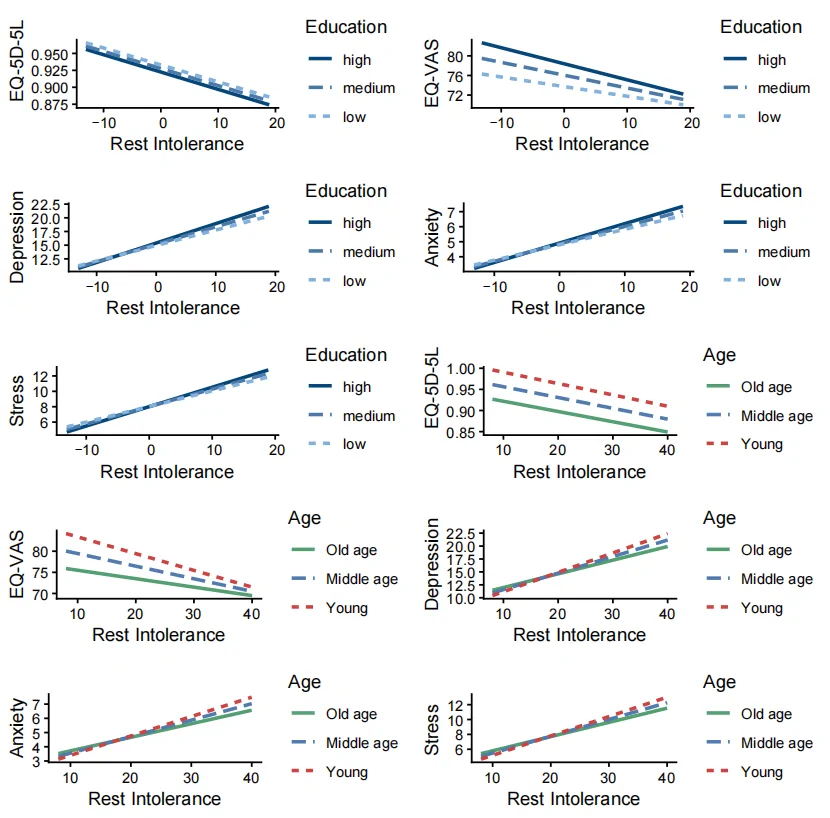
Moderating effects of educational attainment and age on the associations of rest intolerance with quality of life and mental health. EQ-VAS: EuroQol visual analog scale.

## Discussion

### Principal Findings

This study is the first large-scale systematic investigation of the prevalence of rest intolerance among adults in China and its associations with health indicators, with particular attention to its distinct characteristics among highly educated and younger populations. The results indicate that the overall prevalence of high rest intolerance among adults in China is 14.94% (3645/24,405). Younger individuals and those with higher educational attainment exhibit higher levels of rest intolerance. Within these groups, rest intolerance is also more strongly associated with poorer mental health and lower quality of life.

This finding of a 14.94% prevalence of high rest intolerance provides an important epidemiological reference for understanding the distribution of this phenomenon in the general population. Notably, previous research has confirmed the validity of cluster analysis and latent profile analysis in determining scale cutoff points [28]. The cutoff (28 points) derived through cross-validation of these 2 methods demonstrates robust reliability, providing a solid foundation for future related studies. However, it is worth noting that this cutoff is statistically derived and lacks external validation against clinical benchmarks. Consequently, its clinical utility is yet to be established, and we recommend that future research validate its clinical applicability before any clinical adoption.

More notably, this study found significant variations in the distribution of high rest intolerance across different populations, revealing a clear pattern: individuals with higher educational attainment and younger individuals constitute high-risk populations for rest intolerance. Specifically, the prevalence of high rest intolerance showed a marked upward trend with increasing educational attainment, climbing from 8.25% (93/1127) among those with no formal education to 31.29% (87/278) among doctoral degree holders. Age differences were equally pronounced, with young adults exhibiting a high rest intolerance prevalence of 18.68% (1829/9792), significantly higher than that of middle-aged and older groups. It should be noted that both age and education were significantly associated with rest intolerance, yet the associations had modest effect sizes that explain only a small share of the variance. Empirical evidence concerning the sociodemographic distribution of rest intolerance remains limited because the construct has only recently been formally operationalized. A recent 4-study investigation of leisure guilt found that younger adults experienced guilt during leisure more frequently than older adults [29]. This finding is consistent with the age pattern observed in the present study, although leisure guilt captures only 1 component of the broader construct of rest intolerance. A nationwide study of 1878 Chinese nursing students also identified age as a potentially relevant factor [8]. Age ranked fifth among 18 candidate variables in the random forest model, indicating relatively high predictive importance, although its association was not significant in the subsequent univariate generalized linear model. Educational background was included in the random forest analysis but showed lower relative importance and was not carried forward to the regression models. These results provide partial support for the demographic relevance observed in the present study, while differences in analytic methods, age range, and population composition may explain why the specific age and educational gradients were more pronounced in our broader sample. Qualitative evidence further supports several mechanisms that may underlie these demographic patterns. Among nursing interns, rest intolerance manifested as social comparison, productivity-based professional identity, persistent task-related rumination, and difficulty psychologically disengaging during rest [30]. Participants attributed these experiences to transitional stress and an imbalance between available time and perceived demands. These findings suggest that achievement pressure and identity formation may be particularly salient during education and the transition into professional careers.

For highly educated individuals, prolonged periods of intensive study and work may foster a self-identity centered on continuous productivity. Consequently, they may be more likely to perceive rest as a waste of time [12]. Career expectations and social pressures associated with higher educational attainment may also make it difficult to disengage during nonworking hours [3]. Nevertheless, previous studies have not directly established that these mechanisms account for the observed educational gradient, which should therefore be interpreted cautiously. For younger individuals, early career development and upward occupational mobility may expose them to particularly intense competition and achievement pressure. These demands may be compounded by digitally mediated work-life boundaries, through which constant connectivity becomes normalized and psychological disengagement becomes increasingly difficult [6]. Rest may consequently become a source of anxiety rather than recovery. However, given the modest effect sizes and cross-sectional nature of the evidence, age and education should not be regarded as deterministic predictors. Longitudinal and cross-cultural studies are needed to establish whether these subgroup differences are reproducible and to clarify their underlying mechanisms.

Second, this study found that rest intolerance is significantly associated with mental health and quality of life. Regression analysis revealed that rest intolerance was significantly and positively associated with anxiety, depression, and perceived stress, while being significantly and negatively associated with quality of life. These findings suggest that an inability to achieve genuine relaxation and recovery during rest may impair individuals’ overall sense of well-being and quality of life. These findings align with those reported by Zhao et al [8] in a nursing student sample and Türk et al [31] in a Turkish university student sample, suggesting that the negative association between rest intolerance and mental health is observable across the limited populations and cultural contexts examined to date. Furthermore, by broadening the research focus beyond specific professions or student groups to the general adult population, this study addresses a key limitation in existing literature regarding the generalizability of findings.

More importantly, moderation analyses revealed that educational attainment and age not only correlated with rest intolerance levels but also amplified its adverse effects on mental health and quality of life. Specifically, individuals with higher educational attainment and younger adults exhibited stronger associations between rest intolerance and anxiety, depression, perceived stress, and EQ-VAS scores, indicating that these groups are not only at higher levels of rest intolerance but also may be more vulnerable to its detrimental health consequences. This pattern can be understood through the performance internalization model of rest intolerance [12]: highly educated individuals, shaped by prolonged exposure to performance-oriented education, tend to anchor self-worth more heavily on external validation [5], making rest intolerance more likely to trigger intense self-criticism and shame. Younger adults, situated at early career stages, face heightened social comparison and blurred digital work-life boundaries [11], rendering psychological disengagement during rest particularly difficult and amplifying their sensitivity to rest-related distress. Notably, age did not significantly moderate the association between rest intolerance and the EQ-5D-5L utility index. This may reflect the distinct measurement properties of the 2 instruments: although EQ-5D-5L assesses concrete functional dimensions that capture relatively objective health states, EQ-VAS requires a global subjective evaluation of overall health. As a construct centered on negative affect and cognitive bias, rest intolerance may exert a stronger influence on subjective health perceptions than on objective functional limitations, with relatively small age-related variation in the latter. Additionally, the moderating effect of educational attainment on the EQ-5D-5L association became nonsignificant after controlling for chronic disease status, smoking, and alcohol consumption, suggesting that this effect may be mediated by health behaviors or may be inherently modest, warranting further validation.

This study offers multiple insights for public health and clinical practice. First, health education on rest intolerance should be implemented in settings where young and highly educated individuals congregate, such as universities and workplaces, to help individuals recognize the importance of effective rest for long-term health and well-being. Second, psychological interventions should focus on individuals’ cognitive evaluations of rest, helping them alleviate guilt or anxiety associated with taking breaks. Additionally, organizations should advocate for healthy workplace cultures that respect employees’ rest time and reduce work encroachment on nonworking hours.

Finally, this study also has several limitations. First, due to the constraints of the cross-sectional design, it is impossible to confirm a causal relationship between rest intolerance and mental health. We acknowledge that the observed associations could also reflect reverse causation (eg, anxiety or depression leading to rest intolerance) or bidirectional influences. Therefore, future longitudinal or cross-lagged studies are warranted to clarify the temporal and causal dynamics between these constructs. Second, this study primarily relies on self-reported data; subsequent research could incorporate physiological indicators or behavioral observations for multimodal measurement. Third, the cutoff for rest intolerance established in this study requires further validation in prospective research to determine its clinical significance and predictive validity. Finally, although the performance internalization model of rest intolerance was developed with particular attention to the Chinese sociocultural context, where examination-intensive education and strong performance norms are salient, the generalizability of our findings to other cultural or societal settings remains to be established. Future cross-cultural research is needed to examine whether the observed patterns and the proposed model hold similarly in societies with different educational systems, work cultures, or value orientations regarding rest and productivity.

### Conclusions

This study is the first to reveal the prevalence characteristics of rest intolerance among adults in China, clearly identifying highly educated and younger individuals as groups with higher levels of rest intolerance. Rest intolerance is not only more prevalent in these groups but also exhibits a significant negative association with their mental health and quality of life. These findings suggest that educational institutions and workplace organizations should consider this an emerging health concern warranting targeted response.

## Appendix

Multimedia Appendix 1 Validity of the Rest Intolerance Scale–8 (RIS-8) in the full sample.

Multimedia Appendix 2 Measurement invariance testing.

Multimedia Appendix 3 Detailed fit indices for each latent profile model.

Multimedia Appendix 4 Stability analysis with additional covariates.

## Abbreviations

BIC: Bayesian information criterion
ChiCTR: Chinese Clinical Trial Registry
EQ-VAS: EuroQol visual analog scale
GAD-R3: Generalized Anxiety Disorder–R3
PBICR: Psychology and Behavior Investigation of Chinese Residents
PHQ-9: Patient Health Questionnaire–9
PSS-4: Perceived Stress Scale–4
RIS-8: Rest Intolerance Scale–8

## Data Availability

The datasets generated and/or analyzed during the current study are not publicly available but are accessible from the corresponding author upon reasonable request.
