# Supplementary material for: Prevalence of Rest Intolerance and Its Associations With Mental Health and Quality of Life Among Adults in China: Cross-Sectional Study
Source: JMIR Public Health Surveill. 2026 Oct 1;12:e100611. doi: 10.2196/100611 (PMC13673177; doi:10.2196/100611)
Supplement: Supplementary file 1 [file publichealth_v12i1e100611_app1.docx]

**Appendix 1: Validity of the RIS-8 in the Full Sample**

A confirmatory factor analysis (CFA) was conducted on the RIS-8 using the full sample to evaluate its overall psychometric properties.The results supported the hypothesized four-factor structure of the RIS-8, comprising negative affect, social comparison, obsessive thoughts, and cognitive bias.The model demonstrated good fit indices (CFI = 0.99, TLI = 0.98, RMSEA = 0.049), indicating sound construct validity of the scale within this sample of Chinese residents.In summary, the RIS-8 exhibited robust construct validity in the full population sample, thereby providing a reliable measurement foundation for subsequent population-level analyses and group comparisons.
