# Supplementary material for: Prevalence of Rest Intolerance and Its Associations With Mental Health and Quality of Life Among Adults in China: Cross-Sectional Study
Source: JMIR Public Health Surveill. 2026 Oct 1;12:e100611. doi: 10.2196/100611 (PMC13673177; doi:10.2196/100611)
Supplement: Supplementary file 2 [file publichealth_v12i1e100611_app2.docx]

**Appendix 2: Measurement Invariance Testing**

To assess the applicability of the RIS-8 across different demographic groups, multi-group confirmatory factor analysis (MGCFA) was conducted to test for configural, metric, and scalar invariance across age groups, gender, ethnicity, and educational attainment levels.The results indicated that the configural model exhibited good fit across all subgroup conditions (CFI = 0.989–0.990, RMSEA = 0.058–0.070), supporting a consistent factor structure of the RIS-8 across different populations.After imposing equality constraints on factor loadings, the fit indices for the metric invariance model showed minimal change, with ΔCFI values below 0.01 across all subgroup comparisons. This supported metric invariance, indicating that the RIS-8 items measured the latent constructs consistently across groups.In the test for scalar invariance, although the Satorra–Bentler scaled chi-square difference test reached statistical significance in some comparisons, the ΔCFI values remained below 0.01 across all subgroups, meeting the recommended criteria for establishing measurement invariance in contemporary research.Therefore, scalar invariance of the RIS-8 was established across age groups, gender, ethnicity, and educational attainment levels.In summary, the RIS-8 demonstrated satisfactory measurement equivalence across diverse demographic groups within the Chinese adult sample, supporting its use for valid comparisons between different population subgroups.

Supplementary Table 1. Measurement Invariance of the RIS-8 Across Demographic Groups

| Group Variable | Invariance Level | df | χ² | CFI | RMSEA | ΔCFI |
| --- | --- | --- | --- | --- | --- | --- |
| Age Group | Configural | 42 | 1569.8 | 0.990 | 0.067 | — |
|  | Metric | 50 | 1597.1 | 0.989 | 0.062 | -0.001 |
|  | Scalar | 58 | 1721.9 | 0.989 | 0.059 | 0.000 |
| Gender | Configural | 28 | 1471.9 | 0.990 | 0.065 | — |
|  | Metric | 32 | 1491.5 | 0.990 | 0.061 | 0.000 |
|  | Scalar | 36 | 1551.5 | 0.990 | 0.059 | 0.000 |
| Ethnicity | Configural | 28 | 1495.0 | 0.990 | 0.066 | — |
|  | Metric | 32 | 1507.2 | 0.990 | 0.061 | 0.000 |
|  | Scalar | 36 | 1539.3 | 0.990 | 0.058 | 0.000 |
| Educational Attainment | Configural | 126 | 1783.9 | 0.989 | 0.070 | — |
|  | Metric | 158 | 1846.6 | 0.989 | 0.063 | 0.000 |
|  | Scalar | 190 | 2048.7 | 0.987 | 0.060 | -0.002 |
