# Supplementary material for: Prevalence of Rest Intolerance and Its Associations With Mental Health and Quality of Life Among Adults in China: Cross-Sectional Study
Source: JMIR Public Health Surveill. 2026 Oct 1;12:e100611. doi: 10.2196/100611 (PMC13673177; doi:10.2196/100611)
Supplement: Supplementary file 3 [file publichealth_v12i1e100611_app3.docx]

**Appendix 3 Detailed Fit Indices for Each Latent Profiles Model**

Supplementary Table 2 Fit Indices for Latent Profiles Models (1–6 Classes)

| Classes | AIC | BIC | Entropy |
| --- | --- | --- | --- |
| 1 | 598984.851 | 599114.492 | 1 |
| 2 | 511178.97 | 511381.534 | 0.917 |
| 3 | 478627.575 | 478903.061 | 0.913 |
| 4 | 459048.057 | 459396.466 | 0.913 |
| 5 | 452583.378 | 453004.71 | 0.91 |
| 6 | 446759.547 | 447253.802 | 0.912 |

Model fit indices for the latent profiles analysis are presented in Supplementary Table 2. As shown, the AIC and BIC values decreased progressively as the number of classes increased from 1 to 6, indicating improved model fit with additional classes. The entropy values remained consistently high across all class solutions (ranging from 0.910 to 0.917 for Classes 2-6), all exceeding the recommended threshold of 0.80, suggesting excellent classification accuracy. Considering both model parsimony and interpretability, the 6-class solution demonstrated the lowest AIC (446,759.547) and BIC (447,253.802) among all models, with satisfactory entropy (0.912). These results support the selection of the 6-class model as the optimal solution for subsequent analyses.
