# Supplementary material for: Prevalence of Rest Intolerance and Its Associations With Mental Health and Quality of Life Among Adults in China: Cross-Sectional Study
Source: JMIR Public Health Surveill. 2026 Oct 1;12:e100611. doi: 10.2196/100611 (PMC13673177; doi:10.2196/100611)
Supplement: Supplementary file 4 [file publichealth_v12i1e100611_app4.docx]

**Appendix 4: Stability Analysis with Additional Covariates**

To further test the robustness of the results, and given the availability of additional covariates in the PBICR database, we further controlled for the presence of chronic diseases, alcohol consumption, and smoking status, and re-examined both the main effects of rest intolerance on mental health and quality of life, as well as the moderating effects of age and educational level. The results showed that total rest intolerance scores significantly and negatively predicted both the utility index of quality of life (β = -0.113, p < 0.001, 95% CI = [-0.125, -0.100]) and VAS ratings (β = -0.114, p < 0.001, 95% CI = [-0.127, -0.102]). Conversely, rest intolerance significantly and positively predicted levels of anxiety (β = 0.403, p < 0.001, 95% CI = [0.391, 0.415]), depression (β = 0.398, p < 0.001, 95% CI = [0.387, 0.410]), and perceived stress (β = 0.436, p < 0.001, 95% CI = [0.425, 0.448]).

Furthermore, after additionally controlling for chronic disease status, alcohol consumption, and smoking, we examined whether educational level and age moderated the associations between rest intolerance and quality of life and mental health. The results indicated that educational level significantly moderated the relationships between rest intolerance and quality of life (EQ-VAS: t = -2.873, p = 0.004) and mental health (depression: t = 7.31, p < 0.001; anxiety: t = 7.63, p < 0.001; perceived stress: t = 7.99, p < 0.001), whereas the moderating effect of educational level on the association between rest intolerance and the EQ-5D utility index was not significant (t = -1.08, p = 0.282). Similarly, age significantly moderated the relationships between rest intolerance and quality of life (EQ-VAS: t = 5.20, p < 0.001) and mental health (depression: t = -11.65, p < 0.001; anxiety: t = -12.17, p < 0.001; perceived stress: t = -11.43, p < 0.001), but its moderating effect on the association between rest intolerance and the EQ-5D utility index was not significant (t = 0.123, p = 0.902).
